# Artificial intelligence chatbots as information sources for herpes zoster: an evaluation of patient-education suitability, overall quality, and readability

**DOI:** 10.3389/fpubh.2026.1961492

**Published:** 2026-10-27

**Authors:** Fengyu Zhang, Pengfei Tong, Hucai Yang

**Affiliations:** 1 Department of Pharmacy, The Fourth Hospital of Changsha (Integrated Traditional Chinese and Western Medicine Hospital of Changsha), Changsha, China; 2 International Advanced Technology Application Promotion Center (Hefei), Hefei, Anhui, China; 3 Department of Dermatology, Ningbo, China

**Keywords:** global quality score, herpes zoster, large language models, patient education, readability

## Abstract

**Objective:**

Clarify the effects of large-language-model types and health-education content categories on generated-text quality (patient-education suitability, overall quality) and readability, so as to provide empirical evidence for standardized AI-assisted health-communication applications.

**Methods:**

This cross-sectional study was conducted between July 10 and July 15, 2026. Twenty herpes zoster-related questions were developed by a dermatologist and a pharmacist and categorized into five themes. The questions were then entered into five mainstream large language models (GPT-5, Doubao, DeepSeek, Qianwen, and Gemini), generating 100 textual responses. Response quality was assessed using the Chinese version of the Patient Education Materials Assessment Tool for Printable Materials (C-PEMAT-P) and Global Quality Score (GQS). Readability was evaluated via seven indices including the Automated Readability Index (ARI) and Flesch Reading Ease Score (FRES). Readability outcomes were compared using one-way repeated-measures analysis of variance with Greenhouse–Geisser correction. C-PEMAT-P raw totals and ordinal GQS scores were compared using Friedman tests. Post-hoc comparisons were adjusted using the Holm method. Category-level comparisons were performed using linear mixed-effects models with likelihood-ratio χ^2^ tests for continuous outcomes and an ordinal generalized estimating equation with a Wald χ^2^ test for GQS.

**Results:**

Significant overall differences among models were observed for all seven readability indices and both assessment scores (all *p* < 0.001). Median C-PEMAT-P raw total scores were 12.50, 11.00, 10.00, 9.00 and 9.00 for GPT-5, Doubao, DeepSeek, Qianwen and Gemini, respectively; corresponding median GQS scores were 5.00, 4.00, 4.00, 3.00, and 3.00. After Holm correction, GPT-5 achieved higher scores than each of the other models on both assessment measures. However, no significant difference in C-PEMAT-P raw totals was found between DeepSeek and either Qianwen or Gemini, or between Doubao and DeepSeek, after adjustment (all adjusted *p* = 0.062), and Qianwen and Gemini did not differ significantly (adjusted *p* = 0.766). The overall effect sizes were Kendall’s W = 0.611 for C-PEMAT-P and W = 0.779 for GQS. Exploratory pooled Spearman correlation analysis revealed a positive association between the two assessment scores (ρ = 0.853).

**Conclusion:**

Model type is a key determinant of text quality. Readability was independently influenced by both model type and content category, and the two are relatively independent. In clinical applications, GPT-5, DeepSeek, and Doubao are preferred. Readability can be optimized by integrating topic characteristics and patients’ health literacy to enhance communication accuracy. Limitations exist in this study regarding sample coverage and evaluation dimensions (the absence of subjective experience data); larger-scale verification should be conducted in future research.

## Introduction

Herpes zoster (HZ) is an infectious skin disease caused by reactivation of varicella-zoster virus (VZV) that resides latently in the dorsal root or cranial ganglia, a process typically triggered by waning host cellular immunity (1, 2). Clinically, HZ is characterized by unilateral zosteriform vesicles accompanied by neuropathic pain, with a predilection for the older adults and immunocompromised populations. Although the disease itself is rarely fatal, its most common and debilitating sequela is postherpetic neuralgia (PHN)—a persistent pain syndrome that may persist for months or even years. This refractory chronic pain not only disrupts sleep and emotional well-being but can also precipitate suicidal ideation, profoundly diminishing patients’ overall quality of life and imposing substantial economic strain (3, 4). According to global epidemiological surveys, the annual incidence of HZ ranges from 3 to 5 cases per 1,000 individuals, with a steady upward trend of 2.5–5.0% per year (5–7). Notably, the progressive decline in cell-mediated immunity associated with aging, underlying immunosuppressive disorders, or immunosuppressive therapies substantially increases the risks of HZ occurrence, hospitalization, and mortality, thereby presenting persistent and escalating challenges to both healthcare delivery and mental health support systems.

For patients diagnosed with herpes zoster, mainstream clinical interventions primarily consist of antiviral treatment, analgesic management, and corticosteroid therapy, all of which require long-term management and regular follow-up (8–10). For susceptible and uninfected populations, HZ vaccination serves as a valid and effective preventive strategy. Vaccination aims to inhibit VZV reactivation, thereby preventing HZ, PHN, and other virus-related complications. Existing studies have confirmed that patients’ health literacy regarding HZ significantly influences their treatment adherence and prognostic outcomes (11). Accordingly, delivering scientific, accurate, and accessible health education is essential for disease control. Nevertheless, conventional health education methods are limited in coverage and efficiency (12, 13). The popularization of the Internet, as well as the rapid development of online medical platforms and social media, has profoundly reshaped patients’ approaches to acquiring health-related information. However, online medical information is often heterogeneous in quality (14–16). Inaccurate and one-sided information may mislead patients’ medical decisions and negatively affect their psychological status and disease management (17, 18).

Artificial intelligence (AI) technology, especially the rapid advancement of large language models (LLMs) in recent years, has offered novel opportunities for medical science popularization (19, 20). Trained on massive textual datasets, LLMs are able to generate well-structured, logically rigorous responses via natural language interaction. Typical overseas LLMs, represented by ChatGPT, have exhibited promising application potential in medical question answering, patient consultation, and health education (21, 22). Meanwhile, domestic homologous models have also been gradually applied to assist clinicians in clinical decision-making and provide personalized health management guidance. Nevertheless, the implementation of LLMs in medical popularization still confronts multiple challenges, including limited reliability, untimely updates, uneven quality of training data, and inherent annotation difficulties. Substantial differences among various models in linguistic style, text readability, logical rigor, and reference accuracy can directly affect patients’ comprehension of and trust in popular science information (23, 24). Notably, there is currently a lack of systematic evaluation studies focusing on the practical performance of LLMs in herpes zoster-related health popularization.

The present study aimed to compare five large language models—GPT-5, Doubao, DeepSeek, Qianwen, and Gemini—in their responses to standardized patient-education questions about herpes zoster. The evaluation focused on recorded patient-education assessment scores, overall response quality, and readability. These outcomes were examined as distinct dimensions of the generated educational content and were not intended to establish factual accuracy or clinical safety.

## Materials and methods

### Ethical considerations

All data adopted in this study are generated by artificial intelligence models, with no involvement of human or animal experiments. In addition, no personal sensitive information or biological samples from real human subjects are included. In accordance with standardized academic and ethical guidelines, ethical review approval is not required for this type of research.

### Research procedure

A dermatologist and a pharmacist collaboratively compiled 20 common patient-education questions about herpes zoster, covering five core thematic dimensions: basic knowledge, etiology and risk factors, clinical diagnosis, treatment strategies, and prevention and rehabilitation, with detailed classification displayed in Table 1. Each domain contained four questions. The same questions were submitted to five LLMs, yielding 20 responses per model and 100 responses overall.

**Table 1:** Standardized herpes zoster-related questions used in the study.

No.	Health-education content category	Standardized question
1	Basic knowledge	What is herpes zoster, and what are its main pathological features?
2	Basic knowledge	What are the main differences and connections between herpes zoster and chickenpox?
3	Basic knowledge	What are the typical skin symptoms and pain characteristics of active herpes zoster?
4	Basic knowledge	Is herpes zoster contagious, and how can the virus spread?
5	Etiology and risk factors	What virus causes herpes zoster, and how does it become active again in the body?
6	Etiology and risk factors	What are the main risk factors for developing herpes zoster?
7	Etiology and risk factors	How does a weak immune system affect the risk and severity of herpes zoster?
8	Etiology and risk factors	Can close contact with a person who has herpes zoster directly cause herpes zoster, and who may develop chickenpox after contact?
9	Diagnosis	What symptoms and physical signs are commonly used to diagnose herpes zoster?
10	Diagnosis	What is the role of VZV nucleic acid testing of blister fluid or skin samples in diagnosing herpes zoster?
11	Diagnosis	How can herpes zoster be distinguished from herpes simplex, contact dermatitis, and other similar diseases?
12	Diagnosis	How can special types of herpes zoster, such as zoster without a rash, disseminated zoster, eye-related zoster, and ear-related zoster, be identified?
13	Treatment	What antiviral drugs are commonly used as first-line treatments for herpes zoster?
14	Treatment	How soon should antiviral treatment begin after symptoms appear, and how long does treatment usually last?
15	Treatment	What are the main treatment methods for acute herpes zoster pain and postherpetic neuralgia?
16	Treatment	What are the common side effects and precautions of antiviral drugs and medicines used for nerve pain?
17	Prevention and rehabilitation	What measures can reduce the spread of the virus and help patients care for their skin lesions?
18	Prevention and rehabilitation	Who should receive the herpes zoster vaccine, and can it prevent herpes zoster and postherpetic neuralgia?
19	Prevention and rehabilitation	Should people who have already had herpes zoster still receive the vaccine, and when should they receive it?
20	Prevention and rehabilitation	During recovery, how should patients monitor their skin lesions, pain, and treatment progress, and when should they seek medical care?

The experimental unit for model comparisons was the question. Accordingly, the five responses corresponding to the same question were treated as matched observations. Questions were identified by the combination of their educational domain and within-domain question number.

### LLM response generation

Responses were collected July 10–15, 2026, using the official web interfaces of GPT-5, Doubao, DeepSeek, Qianwen and Gemini rather than their application programming interfaces (APIs). The same set of English-language questions was submitted to each model, and the responses included in the readability analysis were in English.

The models were accessed using the settings available through their respective web interfaces. API-level generation parameters, including temperature and top-p, were not available in the study records and were not standardized.

Because the models were accessed through web interfaces rather than APIs, exact model build identifiers, conversation initialization procedures, web-search settings, response-regeneration procedures, and other interface-level settings were not available in the study records. These limitations may affect exact replication of the response-generation process.

### Readability assessment

Given that no single readability metric has been established as a universal gold standard to date, this study adopted a panel of mainstream readability indicators extensively validated in prior relevant studies. Readability was assessed using seven recorded indices: the Automated Readability index (ARI) (25), Simple Measure of Gobbledygook (SMOG) (26), Coleman-Liau Readability Index (CL) (27), Linsear Write (LW) (28), Gunning Fog Readability Index (GFOG) (29), Flesch Reading Ease Score (FRES) (30) and Flesch–Kincaid Grade Level (FKGL) (31). The original assessment used ReadabilityFormulas.com^1^ as the calculation platform. The platform was regarded as a computational tool rather than evidence establishing the validity of the underlying formulas.

The analyzed responses were in English, consistent with the language for which these formulas were developed. Higher FRES values indicate easier reading, whereas higher ARI, GFOG, FKGL, CL, and SMOG values generally indicate greater estimated reading difficulty. These indices quantify selected linguistic characteristics and do not directly measure patient comprehension, factual accuracy, or clinical safety.

The LW values were retained as recorded for exploratory comparison. Because the available records did not establish which LW implementation was used, these values were not interpreted as word counts or assigned specific educational grade levels.

### Quality assessment

The reliability and overall quality of LLM-generated responses were quantitatively assessed using two validated evaluation tools: patient-education suitability metrics (C-PEMAT-P) (32, 33) and the Global Quality Score (GQS) (34). The C-PEMAT-P scale comprises 24 items categorized into two core dimensions: understandability (17 items, covering logical content organization and accessible expression of professional terminology) and actionability (7 items, evaluating operational guidance and applicability for target populations). The GQS is rated on a 1–5 Likert scale, where higher scores indicate better comprehensive content quality (35–37).

The available dataset contained binary item ratings and their sum across the 24C-PEMAT-P items. For transparency, this recorded outcome was analyzed as the C-PEMAT-P raw total, with a possible range of 0–24. It was not interpreted as a standard PEMAT percentage score. Standard PEMAT scoring calculates understandability and actionability separately and excludes nonapplicable items from the relevant denominator. The available dataset did not separately identify nonapplicable items, preventing retrospective verification of those denominators.

On July 10–15, 2026, two pharmacist experts with more than 3 years of relevant professional experience used two scales to evaluate the answers separately. Any scoring discrepancies were adjudicated by a third senior expert and finalized through group consensus discussion (38). The reanalysis used the recorded final scores without modifying individual ratings. Neither C-PEMAT-P nor GQS constituted an independent assessment of factual accuracy or clinical safety against an external clinical reference standard.

### Statistical analysis

Statistical analyses were performed with the question as the matched experimental unit because the same 20 questions were submitted to each of the five large language models. Thus, responses generated for the same question across models were treated as correlated repeated observations rather than independent samples.

Continuous readability measures (ARI, FRES, GFOG, FKGL, CL, SMOG, and LW) were summarized as mean ± SD and compared among the five models using one-way repeated-measures ANOVA. To provide a conservative inference without relying on the assumption of sphericity, Greenhouse–Geisser-adjusted degrees of freedom and *p* values were reported. Significant omnibus tests were followed by two-sided paired t-tests, with Holm adjustment across the 10 model pairs separately for each outcome.

Because the Global Quality Score (GQS) is an ordinal 5-point scale, it was analyzed as an ordinal outcome rather than as a continuous variable. GQS values were summarized as median and interquartile range (IQR) and compared among models using the Friedman test. Given the bounded discrete nature of the C-PEMAT-P raw total, the same conservative nonparametric repeated-measures approach was used for C-PEMAT-P. Significant Friedman tests were followed by pairwise Wilcoxon signed-rank tests with Holm adjustment for multiple comparisons.

Partial eta squared (ηp^2^) was reported as the effect-size measure for repeated-measures ANOVA, and Kendall’s W was reported for Friedman tests. All tests were two-sided, and a Holm-adjusted *p* value < 0.05 was considered statistically significant for pairwise comparisons. For category-level comparisons, responses were pooled across the five models within each content category (*n* = 20 per category). Continuous outcomes (C-PEMAT-P raw totals and readability indices) were analyzed using linear mixed-effects models with question as a random intercept and LLM and content category as fixed effects; the category effect was assessed with a likelihood-ratio χ^2^ test. The ordinal GQS was analyzed using an ordinal generalized estimating equation with question as the clustering variable, content category and LLM as covariates, and an exchangeable working correlation; the category effect was assessed with a Wald χ^2^ test. All statistical analyses and visualization graphs were generated using Python 3.14.

## Results

### Readability and quality analysis

This study systematically investigated the respective effects of LLM types and health education content categories on the readability and comprehensive quality of AI-generated medical popularization texts. Two sets of comparative analyses were conducted in the present research. The first analysis quantitatively compared five mainstream LLMs (GPT-5, Doubao, DeepSeek, Qianwen, and Gemini) in terms of patient-education applicability, overall content quality, and textual readability. The evaluated indicators included the C-PEMAT-P score, GQS score, and seven classic readability metrics (ARI, FRES, GFOG, FKGL, CL, SMOG, and LW). The second analysis assessed variations in the aforementioned indicators across five classified themes of herpes zoster education: basic knowledge, etiology and risk factors, clinical diagnosis, treatment strategies, and prevention and rehabilitation. Based on the integrated multi-dimensional analytical results, this study further explored the inherent influence patterns of model attributes and content categories on the textual quality and readability of LLM-produced health education materials.

### Comparison of readability and quality measures among LLMs

Because the same 20 questions were evaluated across all five LLMs, between-model comparisons were performed using repeated-measures methods.

Significant overall model effects were observed for all seven readability indices after Greenhouse–Geisser correction, namely ARI (*F* = 18.11, adjusted df = 2.62, 49.81, *p* < 0.001, ηp^2^ = 0.488), FRES (*F* = 26.64, adjusted df = 3.29, 62.57, *p* < 0.001, ηp^2^ = 0.584), GFOG (*F* = 26.59, adjusted df = 3.17, 60.16, *p* < 0.001, ηp^2^ = 0.583), FKGL (*F* = 22.81, adjusted df = 2.87, 54.58, *p* < 0.001, ηp^2^ = 0.546), CL (*F* = 16.17, adjusted df = 3.36, 63.91, *p* < 0.001, ηp^2^ = 0.460), SMOG (*F* = 30.57, adjusted df = 2.92, 55.46, *p* < 0.001, ηp^2^ = 0.617), and LW (*F* = 13.39, adjusted df = 3.16, 60.02, *p* < 0.001, ηp^2^ = 0.413) (Table 2).

**Table 2:** Overall repeated-measures comparisons across five LLMs.

Outcome	Statistical test	Statistic	df	*p* value	Effect size
ARI	RM-ANOVA, GG corrected	*F* = 18.106	2.622, 49.810	1.43 × 10^−7^	ηp^2^ = 0.488
FRES	RM-ANOVA, GG corrected	*F* = 26.639	3.293, 62.570	9.30 × 10^−12^	ηp^2^ = 0.584
GFOG	RM-ANOVA, GG corrected	*F* = 26.594	3.166, 60.155	2.26 × 10^−11^	ηp^2^ = 0.583
FKGL	RM-ANOVA, GG corrected	*F* = 22.811	2.873, 54.580	1.69 × 10^−9^	ηp^2^ = 0.546
CL	RM-ANOVA, GG corrected	*F* = 16.167	3.364, 63.914	2.05 × 10^−8^	ηp^2^ = 0.460
SMOG	RM-ANOVA, GG corrected	*F* = 30.573	2.919, 55.461	1.20 × 10^−11^	ηp^2^ = 0.617
LW	RM-ANOVA, GG corrected	*F* = 13.395	3.159, 60.020	5.59 × 10^−7^	ηp^2^ = 0.413
C-PEMAT-P raw total	Friedman test	χ^2^ = 48.863	4	6.24 × 10^−10^	W = 0.611
GQS	Friedman test	χ^2^ = 62.294	4	9.55 × 10^−13^	W = 0.779

RM-ANOVA, repeated-measures analysis of variance; GG, Greenhouse–Geisser. For RM-ANOVA, numerator and denominator degrees of freedom and p values are GG adjusted. Friedman statistics are corrected for ties. Effect sizes are partial eta squared for RM-ANOVA and Kendall’s W for Friedman tests.

Mean FRES values were 47.35 ± 10.66 for GPT-5, 32.65 ± 11.63 for Doubao, 45.40 ± 17.42 for DeepSeek, 21.95 ± 14.26 for Qianwen, and 39.30 ± 16.08 for Gemini (Table 3). After Holm adjustment, Qianwen had lower FRES values than each of the other four models, whereas GPT-5 had higher FRES values than Doubao and Qianwen but did not differ significantly from DeepSeek or Gemini. The pattern of significant pairwise comparisons varied across the remaining indices; for example, GPT-5 had lower ARI and FKGL values than Doubao, Qianwen, and Gemini, while its differences from DeepSeek were not statistically significant. Complete adjusted pairwise *p* values are provided in Table 4.

**Table 3:** Descriptive statistics of the recorded assessment scores and readability outcomes across five LLMs.

Outcome	GPT-5 (*n* = 20)	Doubao (*n* = 20)	DeepSeek (*n* = 20)	Qianwen (*n* = 20)	Gemini (*n* = 20)
ARI	12.28 ± 1.88	15.98 ± 2.82	14.62 ± 5.02	19.56 ± 3.18	15.64 ± 4.23
FRES	47.35 ± 10.66	32.65 ± 11.63	45.40 ± 17.42	21.95 ± 14.26	39.30 ± 16.08
GFOG	10.47 ± 1.98	12.98 ± 1.98	11.16 ± 3.02	15.21 ± 2.33	12.04 ± 2.49
FKGL	10.98 ± 1.60	14.31 ± 2.20	12.59 ± 3.90	17.24 ± 2.70	13.30 ± 3.40
CL	12.88 ± 2.02	14.80 ± 2.00	12.64 ± 2.91	15.85 ± 2.43	14.04 ± 2.50
SMOG	9.22 ± 1.56	11.79 ± 1.83	10.20 ± 2.95	14.20 ± 2.30	11.00 ± 2.51
LW^1^	61.80 ± 6.41	55.45 ± 6.35	61.10 ± 7.33	52.15 ± 5.17	58.85 ± 7.31
C-PEMAT-P raw total^2^	12.50 (11.00–14.00)	11.00 (9.75–11.00)	10.00 (9.00–10.00)	9.00 (8.00–9.00)	9.00 (8.00–9.00)
GQS	5.00 (4.00–5.00)	4.00 (3.00–4.00)	4.00 (3.75–4.00)	3.00 (2.00–3.00)	3.00 (2.75–3.00)

Readability outcomes are presented as mean ± standard deviation. Assessment scores are presented as median (Q1–Q3), with quartiles calculated using linear interpolation.

^1^LW values are reported as recorded; their implementation and grade-level interpretation were not independently verified.

^2^The recorded sum of 24 binary items, with a possible range of 0–24. This is not a standard PEMAT domain-specific percentage score.

**Table 4:** Pairwise post-hoc comparisons of C-PEMAT-P and GQS among the five LLMs.

Outcome	DB–DS	DB–QW	DB–GM	DB–GPT	DS–QW	DS–GM	DS–GPT	QW–GM	QW–GPT	GM–GPT
ARI	0.612	<0.001	0.669	<0.001	0.001	0.612	0.166	0.005	<0.001	0.009
FRES	0.004	0.002	0.095	<0.001	<0.001	0.174	0.534	<0.001	<0.001	0.058
GFOG	0.016	<0.001	0.162	<0.001	<0.001	0.211	0.291	<0.001	<0.001	0.063
FKGL	0.163	<0.001	0.288	<0.001	<0.001	0.364	0.196	<0.001	<0.001	0.016
CL	0.008	0.140	0.274	0.004	<0.001	0.080	0.616	<0.001	<0.001	0.080
SMOG	0.023	<0.001	0.215	<0.001	<0.001	0.215	0.215	<0.001	<0.001	0.009
LW	0.016	0.061	0.278	<0.001	<0.001	0.420	0.694	0.003	<0.001	0.319
C-PEMAT-P raw total	0.062	0.002	0.002	0.008	0.062	0.062	0.002	0.766	<0.001	0.002
GQS	1.000	0.002	0.002	0.003	0.002	0.002	0.002	1.000	<0.001	<0.001

DB, Doubao; DS, DeepSeek; QW, Qianwen; GM, Gemini; GPT, GPT-5.

Readability outcomes were compared using two-sided paired t-tests. Assessment scores were compared using two-sided Wilcoxon signed-rank tests with tie-adjusted normal approximation, exclusion of zero differences, and no continuity correction. Holm adjustment was applied to the full set of 10 comparisons within each outcome, encompassing both panels. Values are rounded; significance was determined using unrounded adjusted *p* values.

C-PEMAT-P raw totals differed significantly among the five models (Friedman χ^2^ = 48.86, df = 4, *p* < 0.001; Kendall’s W = 0.611) (Table 2). Median raw totals were 12.5 (11.00–14.00) for GPT-5, 11.0 (9.75–11.00) for Doubao, 10.0 (9.00–10.00) for DeepSeek, 9.0 (8.00–9.00) for Qianwen, and 9.0 (8.00–9.00) for Gemini (Table 3). After Holm adjustment, GPT-5 had higher C-PEMAT-P raw totals than each other model, and Doubao also scored higher than Qianwen and Gemini. However, the differences between Doubao and DeepSeek, between DeepSeek and Qianwen, and between DeepSeek and Gemini were not statistically significant after adjustment (all adjusted *p* = 0.062), and Qianwen and Gemini did not differ significantly (adjusted *p* = 0.766).

Similarly, GQS differed significantly among the five models (Friedman χ^2^ = 62.29, df = 4, *p* < 0.001; Kendall’s W = 0.779). Median GQS values were 5.0 (4.00–5.00) for GPT-5, 4.0 (3.00–4.00) for Doubao, 4.0 (3.75–4.00) for DeepSeek, 3.0 (2.00–3.00) for Qianwen, and 3.0 (2.75–3.00) for Gemini (Table 2). GPT-5 scored higher than each other model, and Doubao and DeepSeek each scored higher than Qianwen and Gemini. No significant difference was observed between Doubao and DeepSeek or between Qianwen and Gemini.

These comparisons concern the recorded assessment scores and do not establish differences in independently verified factual accuracy or clinical safety. Moreover, the significant omnibus effects should not be interpreted as evidence that every pair of models differed significantly; complete pairwise comparisons are presented in Table 4.

### Comparison of quality and readability measures across content categories

Because each educational domain comprised four questions, category-level analyses were conducted at the response level (n = 20 per category; five LLMs × four questions). Readability differed significantly across the five content themes with a consistent gradient: basic knowledge texts presented the highest readability (median FRES 51.00, ARI 12.02, CL 11.98), whereas treatment strategy texts exhibited the highest linguistic difficulty (median FRES 28.50, ARI 17.02, CL 15.86), with diagnosis and etiology texts at intermediate levels (FRES χ^2^ = 16.04, *p* = 0.003; the remaining readability indices showed consistent significant differences, all *p* < 0.05; Table 5). In contrast, the long-word proportion index (LW) did not differ significantly across categories (χ^2^ = 7.26, *p* = 0.123), and basic knowledge texts, if anything, contained the highest proportion of long words (median 61.50).

**Table 5:** Comparison across health-education content categories.

Variables	Total (*n* = 100)	Basic knowledge (*n* = 20)	Etiology and risk factors (*n* = 20)	Diagnosis (*n* = 20)	Treatment (*n* = 20)	Prevention and rehabilitation (*n* = 20)	χ^2^	*p*
C-PEMAT-P score, Median [Q1–Q3]	9.50 (9.00, 11.00)	9.00 (8.00–11.00)	10.00 (9.00–11.00)	9.50 (9.00–11.00)	9.50 (9.00–11.00)	10.00 (9.00–12.00)	21.13	<0.001
GQS score, M (Q1, Q3)	3.00 (3.00, 4.00)	3.00 (3.00, 4.00)	3.50 (3.00, 4.00)	3.50 (3.00, 4.00)	3.50 (3.00, 4.00)	4.00 (3.00, 4.00)	13.31	0.010
ARI, M (Q1, Q3)	14.52 (12.62, 18.33)	12.02 (10.12, 14.18)	15.17 (12.37, 18.57)	16.93 (12.78, 19.21)	17.02 (14.40, 19.69)	14.11 (13.17, 18.18)	13.39	0.010
FRES, M (Q1, Q3)	38.00 (26.75, 50.00)	51.00 (41.75, 59.25)	33.50 (24.25, 47.00)	33.50 (20.50, 39.75)	28.50 (14.75, 32.75)	44.00 (34.00, 53.75)	16.04	0.003
GFOG, M (Q1, Q3)	12.30 (10.28, 14.22)	9.65 (8.57, 11.60)	12.20 (10.78, 14.10)	13.95 (12.28, 15.45)	14.15 (11.93, 15.55)	12.00 (10.20, 12.97)	12.86	0.012
FKGL, M (Q1, Q3)	13.27 (10.84, 16.36)	10.57 (8.96, 12.78)	13.57 (11.10, 16.40)	14.98 (12.39, 17.04)	14.95 (12.52, 17.42)	12.09 (10.80, 15.39)	15.35	0.004
CL, M (Q1, Q3)	14.04 (12.13, 15.76)	11.98 (10.46, 12.82)	14.23 (12.60, 16.50)	14.26 (12.65, 15.91)	15.86 (15.14, 17.56)	13.38 (12.60, 14.88)	14.95	0.005
SMOG, M (Q1, Q3)	11.09 (9.05, 13.01)	8.48 (7.58, 10.71)	10.93 (9.54, 12.31)	12.26 (10.65, 14.22)	12.89 (11.15, 14.54)	10.46 (8.76, 11.69)	17.50	0.002
LW, M (Q1, Q3)	57.00 (53.00, 62.25)	61.50 (56.75, 65.25)	55.50 (47.75, 58.00)	57.00 (55.50, 62.00)	56.50 (51.75, 60.50)	59.50 (51.50, 65.75)	7.26	0.123

Data are presented as median (Q1, Q3) for C-PEMAT-P, GQS, and readability indices For category-level comparisons, continuous outcomes were analyzed using linear mixed-effects models with question as a random intercept and LLM and content category as fixed effects, with category effects assessed by likelihood-ratio χ^2^ tests; GQS was analyzed using an ordinal generalized estimating equation with question as the clustering variable, content category and LLM as covariates, and an exchangeable working correlation, with the category effect assessed by a Wald χ^2^ test. All tests were two-sided.

Category effects on core quality indicators, by comparison, were smaller in magnitude and non-monotonic in direction. Although C-PEMAT-P and GQS also differed significantly across categories (C-PEMAT-P χ^2^ = 21.13, *p* < 0.001; GQS χ^2^ = 13.31, *p* = 0.010), the highest-scoring content was not the most readable basic knowledge texts but rather clinically actionable content, namely prevention and rehabilitation (C-PEMAT-P 10.00 (9.00–12.00); GQS 4.00) and treatment (C-PEMAT-P 9.50 (9.00–11.00); GQS 3.50), with an inter-category C-PEMAT-P range of only 1.45 points on the 24-point scale. Because each category comprised only four distinct questions, these category-level comparisons were considered exploratory and should be interpreted with caution.

### Exploratory correlation analysis

The pooled Spearman correlation between the C-PEMAT-P raw total and GQS was 0.853. Correlations between GQS and the seven readability indices ranged from −0.381 to 0.283, whereas correlations between C-PEMAT-P raw totals and readability indices ranged from −0.221 to 0.142 (Supplementary Table S1).

Readability indices were strongly intercorrelated in several instances, including ARI and FKGL (ρ = 0.962) and FRES and GFOG (ρ = −0.906). These associations may partly reflect overlapping linguistic inputs to the formulas.

Because the pooled analysis did not separate within-model, between-model, or question-level associations, the coefficients were interpreted descriptively. They do not establish causal relationships or demonstrate statistical independence between readability and response quality.

### Quality analysis

Figure 1 shows the distribution of the Global Quality Score (GQS) among the five models. GPT-5 achieved the highest GQS scores, with a median of 5.0 (IQR 4.00–5.00); its first quartile of 4.0 indicates that at least 75% of its responses scored 4.0 or above. Statistical analysis confirmed that GPT-5 performed significantly better than each of the other four models (all *p* < 0.01). The boxplot results further indicated that GPT-5 consistently produced high-quality texts without abnormal values, reflecting its stability in generating reliable and standardized health education content.

**Figure 1:**
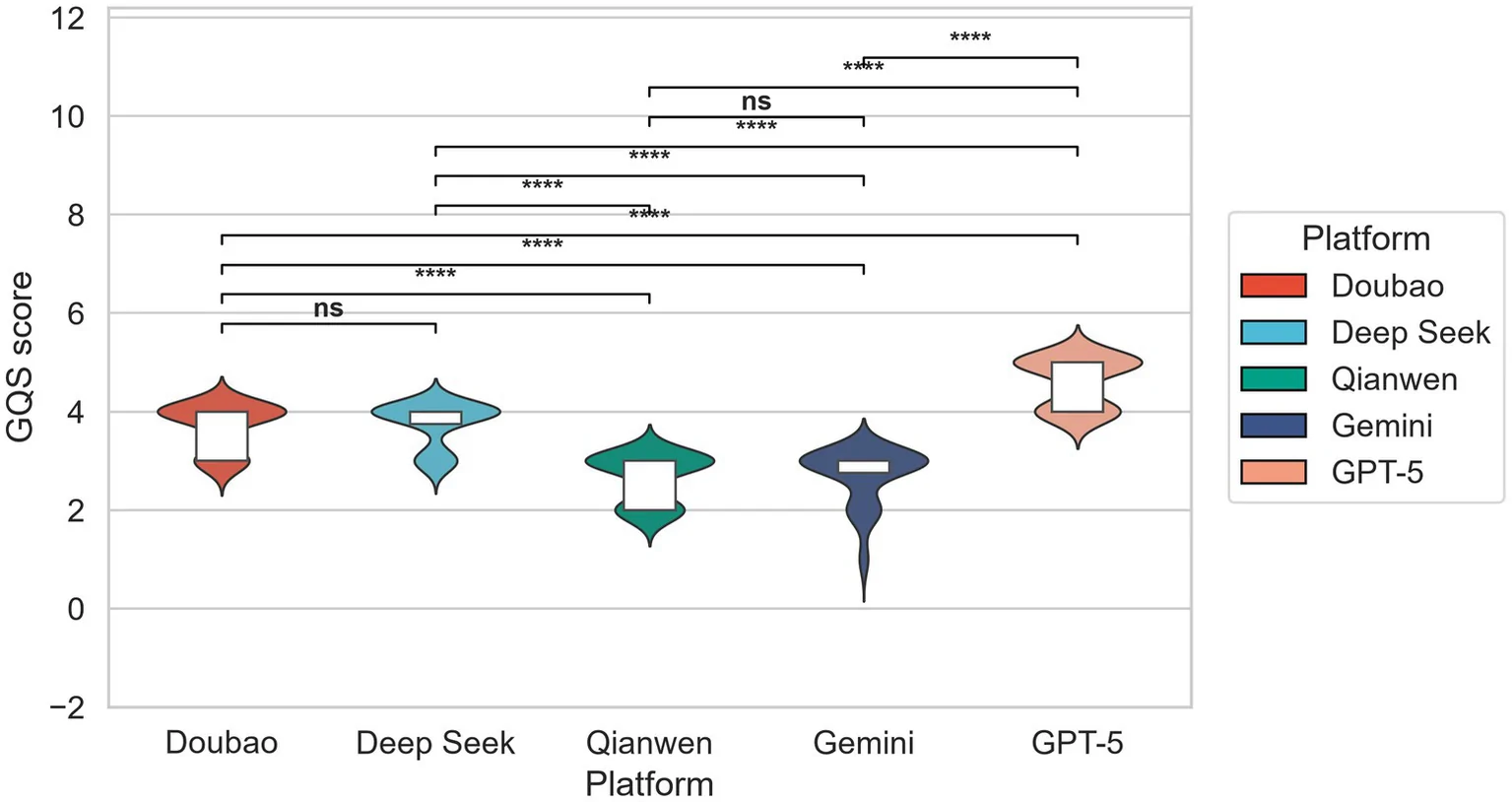
Global Quality score of different large language models.

Doubao and DeepSeek shared the same median score of 4.0, but their distributions differed: DeepSeek showed a highly concentrated distribution (IQR 0.25; interquartile range 3.75–4.00), indicating more stable output quality, whereas Doubao showed a wider spread (IQR 1.0; 3.00–4.00). Nevertheless, the difference between the two models was not statistically significant.

By comparison, Qianwen and Gemini obtained lower GQS scores, with a median of 3.0 for both models. Qianwen showed a broader distribution (IQR 1.0; 2.00–3.00), whereas Gemini was concentrated around 3.0 (IQR 0.25; 2.75–3.00) but exhibited a pronounced, long, narrow downward tail extending toward low scores. This tail suggests unstable overall quality: Gemini was more likely to produce low-quality answers and showed obvious shortcomings in information accuracy and content standardization when generating popular science texts on herpes zoster. Consistent with the statistical results, both Doubao and DeepSeek scored significantly higher than Qianwen and Gemini, whereas no significant difference was observed between Qianwen and Gemini.

As shown in Figure 2, C-PEMAT-P raw total scores varied significantly across models; full test statistics, median values, and Holm-adjusted pairwise comparisons are presented in the preceding model-comparison subsection and in Table 2. These findings indicate that GPT-5-generated texts achieved the highest understandability (clarity of information delivery) and actionability (feasibility of practical guidance) in patient education settings, best matching patients’ cognitive needs and health literacy, followed by Doubao and DeepSeek. Qianwen and Gemini yielded the lowest scores, with narrow violin-plot distributions concentrated in the low-score region, reflecting evident shortcomings in information simplification and clarity of operational guidance; their generated content tended to overuse professional terminology or present vague nursing steps, which may hinder patients’ comprehension and practice of health knowledge.

**Figure 2:**
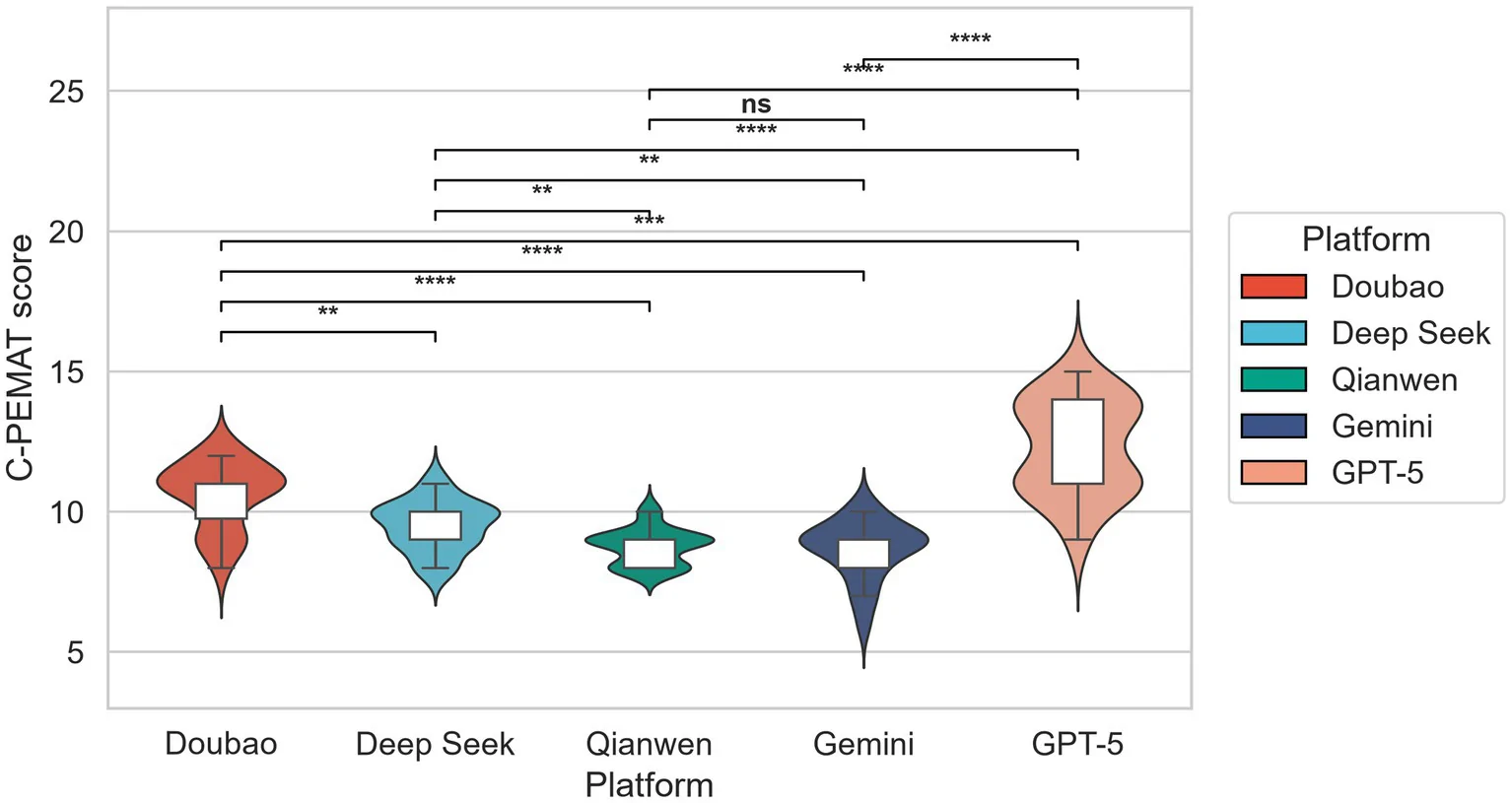
C-PEMAT-P scores of different large language models.

### Correlation analysis

The correlation heatmap in Figure 3 shows the relationships between the quality indicators and the readability indicators. Overall, the correlations between the two types of indicators were relatively weak, but several meaningful trends were identifiable. The GQS score showed a weak positive correlation with the Linsear Write index (LW) (ρ = 0.26), suggesting that text length may serve as a minor factor affecting the overall quality of educational texts. Meanwhile, GQS was weakly negatively correlated with the SMOG index, which evaluates lexical complexity (ρ = −0.34), indicating that increased linguistic complexity did not improve text quality; on the contrary, higher vocabulary difficulty was slightly associated with lower overall quality.

**Figure 3:**
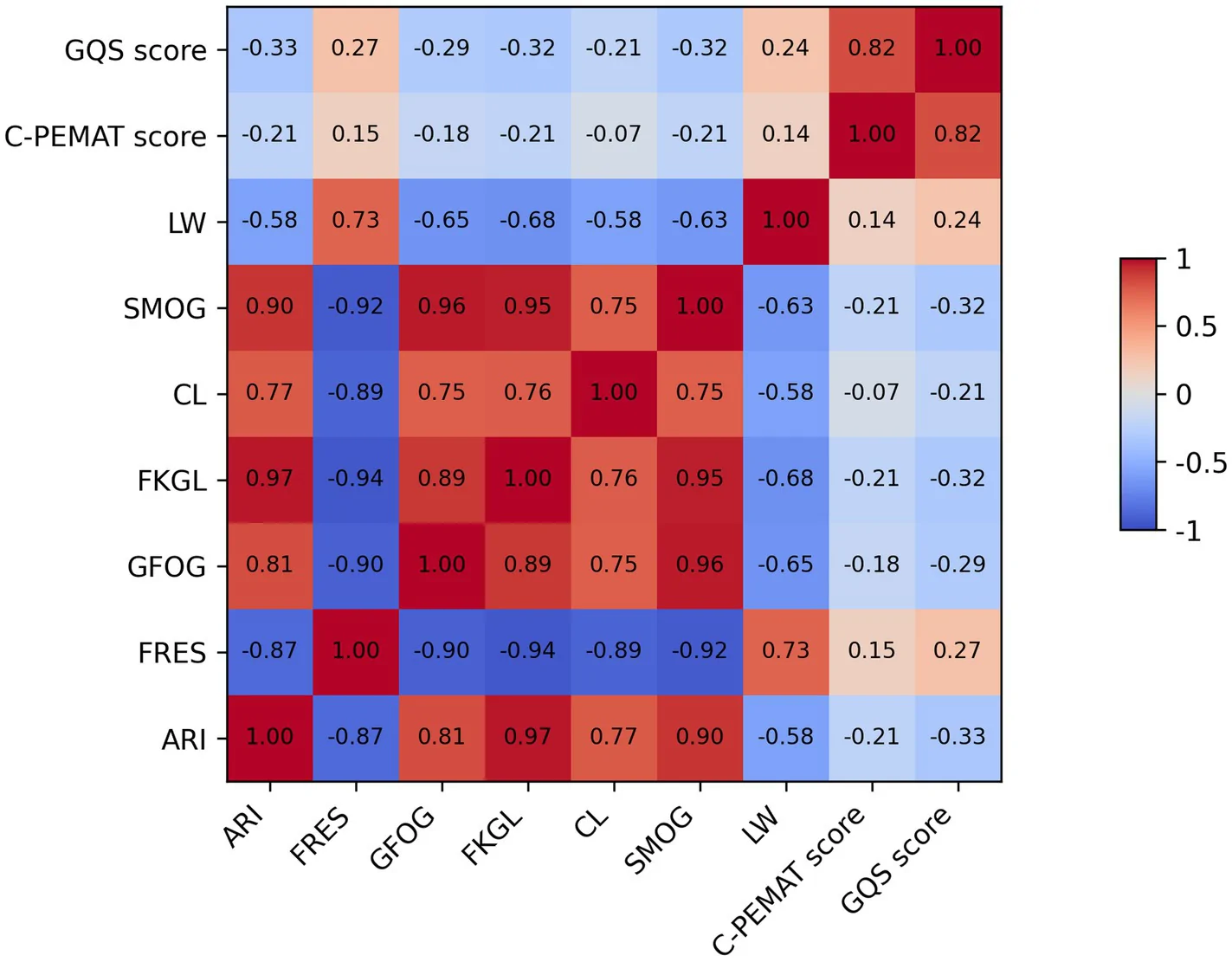
Heatmap of correlations among indicators of large language models.

With respect to the C-PEMAT-P raw total, which reflects patient-education suitability, a strong positive correlation was observed between the C-PEMAT-P raw total and the GQS score (pooled Spearman ρ = 0.85). This finding demonstrated high consistency between the two evaluation dimensions of educational applicability and overall text quality. In contrast, the C-PEMAT-P raw total showed only weak correlations with most readability indicators, with coefficients ranging from −0.22 to 0.14. Specifically, the correlation coefficient between the C-PEMAT-P raw total and LW was 0.14, and that with SMOG was −0.19. These low correlation values indicated that text length and lexical complexity exerted limited direct influence on the suitability of health texts for patient education. The weak negative correlation with SMOG further suggested that overly complex vocabulary may slightly reduce the practical suitability of popular science content, which is consistent with the clinical viewpoint that excessive use of professional terminology hinders patients’ effective understanding and learning of health information.

In contrast, prominent strong correlations were observed among the readability indicators themselves, including ARI and FKGL (ρ = 0.96) and FRES and GFOG (ρ = −0.91). These close correlations indicated that the readability indices complement one another in comprehensively evaluating the overall reading difficulty of educational texts. However, these linguistic indicators showed limited direct influence on text quality. The above results further support that text quality and text readability are two relatively independent evaluation dimensions: text quality primarily depends on information accuracy, content rationality, and logical presentation, whereas text readability is determined primarily by linguistic features, including vocabulary difficulty, sentence structure, and text length.

## Discussion

This study selected model type and health education content category as two core independent variables and systematically analyzed the textual quality and readability characteristics of LLM-generated herpes zoster health education texts based on multi-dimensional evaluation indicators including C-PEMAT-P, GQS, and seven classic readability metrics. Different from previous studies that evaluated readability and content quality simultaneously without distinguishing their independent roles (39, 40), the present study further clarified their independent influencing mechanisms. The overall results demonstrated that model type serves as the dominant determinant of comprehensive text quality and patient education suitability. Textual readability, by contrast, was independently influenced by both model type and content category: the between-model differences in readability were comparable in magnitude to the between-category differences (e.g., an inter-model FRES range of 25.4 vs. an inter-category range of 22.5), with content category exerting a distinct, non-monotonic regulatory effect on readability but only a limited influence on core quality indicators. Correlation analysis further verified that text quality and readability belong to relatively independent evaluation dimensions with weak mutual association. These refined findings provide solid theoretical evidence and targeted practical optimization strategies for the standardized generation and clinical application of high-quality AI patient education texts.

### Determinant role and mechanisms analysis of model type on text quality

At the model level, significant hierarchical differences were observed in the comprehensive quality of patient education texts generated by mainstream LLMs. GPT-5 exhibited prominent advantages in content standardization, patient-oriented suitability and output stability, followed by Doubao and DeepSeek, while Qianwen and Gemini consistently maintained inferior quality performance. Statistical verification confirmed that GPT-5, Doubao, and DeepSeek achieved significantly higher GQS scores than Qianwen and Gemini. For C-PEMAT-P raw totals, GPT-5 and Doubao scored significantly higher than Qianwen and Gemini, whereas DeepSeek did not differ significantly from them after Holm adjustment (all adjusted *p* = 0.062), which is consistent with previous studies demonstrating that high-end closed-source models possess stronger clinical knowledge reasoning and medical text generation capabilities (41, 42). Such inter-model quality discrepancies are not random but essentially reflect the differences in medical domain adaptation, patient-oriented training optimization, and knowledge alignment degree among various LLMs.

The superior quality performance of high-ranking models can be reasonably explained by their training data characteristics and algorithm optimization mechanisms. GPT-5 has been trained on massive standardized patient education corpus and undergone targeted optimization for medical popular science scenarios, enabling it to accurately grasp patient cognitive characteristics and produce content with high understandability and actionability. Similarly, Doubao and DeepSeek have accumulated long-term iterative training in health education fields, forming stable narrative logic and patient-friendly expression habits. In contrast, Qianwen and Gemini lack sufficient targeted medical fine-tuning and patient scenario adaptation, resulting in unstable clinical knowledge output and occasional non-standard medical descriptions, which directly leads to lower C-PEMAT-P and GQS scores. The long downward tail of Gemini’s GQS distribution further indicates that its medical knowledge reasoning has prominent randomness and hallucination risks, a common defect observed in general large models lacking medical domain constraint (43).

Notably, the consistent quality ranking verified by two independent evaluation tools (C-PEMAT-P and GQS) confirms that model-level quality differences are inherent and robust, rather than evaluation scale errors. This study further supplements existing LLM medical evaluation research: previous studies mostly focused on single-index quality comparison, while this study proves that model adaptation to patient education scenarios stably determines the overall quality level of generated texts. Clinically, considering that herpes zoster patients are mostly older adults with limited health literacy, the significant model heterogeneity observed in this study has strong practical guiding significance. It is necessary for medical staff to select high-adaptation LLMs for patient education content generation and conduct professional secondary review to avoid misleading patients caused by low-quality AI output.

### Regulatory effect of content category on text readability and its clinical implications

Content category analysis (Table 5) showed that, unlike its limited and non-monotonic effect on text quality, health education content category mainly regulated textual readability, presenting a pattern of “relatively stable quality but variable readability”. Readability indices showed consistent descriptive gradients across content themes: basic knowledge texts presented the highest readability (median FRES 51.00, ARI 12.02, CL 11.98), whereas clinical diagnosis and treatment strategy texts had substantially higher linguistic difficulty (treatment: median FRES 28.50, ARI 17.02, CL 15.86; all *p* < 0.05; Table 5); only the long-word proportion index (LW) did not differ significantly across categories (χ^2^ = 7.26, *p* = 0.123). This thematic difference is intrinsically determined by the professional attributes of medical content itself rather than model generation capability (44). Basic popular science content mainly introduces disease concepts and epidemiological characteristics, which can be expressed through daily and plain language; in comparison, diagnosis and treatment content involves professional knowledge such as disease pathogenesis, clinical guidelines, and drug intervention mechanisms, which inevitably increases sentence complexity and vocabulary difficulty, thereby reducing overall readability.

In contrast, the regulatory effect of content category on core quality indicators was limited in magnitude and non-monotonic in direction. Although C-PEMAT-P and GQS also differed significantly across categories (χ^2^ = 21.13, *p* < 0.001; χ^2^ = 13.31, *p* = 0.010), the highest-scoring content was not the most readable basic knowledge texts but rather clinically actionable content, namely prevention and rehabilitation (C-PEMAT-P 10.65 ± 2.21) and treatment (10.25 ± 1.83), with an inter-category range of only 1.45 points on a 24-point scale—far smaller than the between-model differences (Figure 2). This pattern supports the relative independence of text quality and readability. High-performance models represented by GPT-5, Doubao, and DeepSeek maintained stable content quality and patient education suitability when facing health education content with different professional complexities. Even for high-difficulty diagnosis and treatment topics, these models optimized content structure and expression logic, adopted hierarchical explanation and targeted popular science strategies, and avoided insufficient information or excessive simplification, with quality not systematically deteriorating as readability declined. This finding is consistent with randomized evidence showing that lowering reading grade level alone does not improve patients’ knowledge acquisition or perceived quality of health information (45).

This category-dependent regulation of text features, especially readability, provides important clinical application value for AI-assisted health education. Medical institutions can flexibly deploy LLM-generated education resources according to patients’ personalized learning needs and disease stages, covering basic popular science, early diagnosis guidance, standardized treatment interpretation, and rehabilitation prevention education, while matching the expected category-related readability profiles to patients’ health literacy. Because high-performance models maintained stable patient education suitability across all content domains, clinicians can greatly improve the efficiency and standardization of clinical patient education at a relatively low cost of quality fluctuation. Nevertheless, limited by the small sample size of each content domain and the single disease scenario of herpes zoster, the universal applicability of this conclusion needs further verification in subsequent multi-disease studies.

### Correlation between text quality and readability and its optimization pathways

The correlation results of this study systematically correct the widespread misconception that “higher readability equals better educational quality” in current AI medical text research. Both global quality (GQS) and patient education suitability (C-PEMAT-P) showed only weak correlation with multi-dimensional readability indicators, confirming that text quality and readability are two relatively independent evaluation dimensions governed by different logical mechanisms. Text quality depends on medical factual accuracy, content completeness and clinical normative rationality, while readability is determined by superficial linguistic features such as text length, sentence structure and vocabulary complexity (46).

Representative correlation trends revealed detailed interaction characteristics between the two dimensions. The weak positive correlation between GQS and the Linsear Write index (LW, ρ = 0.26) indicated that appropriate text length helps improve information integrity and overall quality, while excessive pursuit of brevity may lead to missing key educational information. Meanwhile, the weak negative correlation between GQS and lexical complexity (SMOG, ρ = −0.34) suggested that excessive professional terminology without plain-text interpretation will increase reading barriers and reduce the practical clinical value of high-quality medical content, which is consistent with real patient health education scenarios (44).

In addition, the high internal correlation among readability indices (ARI–FKGL, ρ = 0.962; FRES–GFOG, ρ = −0.906) demonstrated that traditional readability formulas have highly overlapping evaluation dimensions and can jointly reflect textual linguistic difficulty, but they cannot effectively predict the clinical quality and educational effectiveness of AI-generated texts. This further explains why many previous studies had contradictory evaluation results: high readability does not guarantee accurate medical content, and low readability does not represent unqualified educational value.

The independent relationship between quality and readability proposed in this study provides clear dual optimization pathways for subsequent LLM medical scenario iteration and clinical promotion. At the model optimization level, LLMs should maintain high-quality medical knowledge output while adopting adaptive linguistic optimization strategies: professional terminology should be supplemented with plain-language interpretation instead of blind simplification, and high-complexity diagnosis and treatment content should adopt structured and modular expression to balance professionalism and readability. At the clinical application level, medical staff should comprehensively consider dual dimensions of quality and readability, and select personalized AI education materials according to patients’ age structure, health literacy level and disease stage, so as to maximize the effectiveness of intelligent patient education services.

## Limitations

Several limitations should be acknowledged. First, the study included 100 model-generated responses derived from only 20 questions submitted to five LLMs. The limited question sample constrains generalizability beyond the evaluated topics and prompts.

Second, the available records did not permit complete verification of model versions, conversation settings, web-search use, or response-regeneration procedures. These omissions limit exact replication and the attribution of results to specific model configurations.

Third, readability was evaluated using recorded formula-based outputs rather than direct measures of patient comprehension. The original response texts and computational settings were not available for independent recalculation during this reanalysis. In particular, the implementation underlying the recorded LW values could not be verified.

Fourth, the dataset contained C-PEMAT-P raw totals rather than the standard separate percentage scores for understandability and actionability. Nonapplicable items were not separately identified. Furthermore, the Chinese instrument’s validation does not establish equivalent measurement performance when it is applied to English-language responses. These limitations restrict interpretation of the C-PEMAT-P findings.

Fifth, the study did not independently evaluate factual correctness, completeness, harmful misinformation, concordance with authoritative clinical guidelines, or clinical safety. Neither GQS nor the recorded C-PEMAT-P score should be interpreted as direct evidence of these outcomes.

Finally, the analysis did not assess variation across repeated generations, subsequent model updates, patient experience, or clinical outcomes. Pooled correlations were exploratory, and category-level comparisons, based on only four distinct questions per category, were also exploratory and should be interpreted with caution.

## Future directions

Based on the findings and inherent limitations of the present study, several targeted directions are proposed for future research in LLM-based medical patient education. First, subsequent studies should expand the sample volume and disease coverage, adopt standardized scoring protocols for C-PEMAT-P, and record complete model parameters and generation procedures to improve result reproducibility and generalizability. Second, future evaluations should incorporate independent clinical validation, including factual accuracy checking, guideline concordance assessment, and clinical safety verification, rather than relying solely on computational readability and scale-based quality indicators. Third, to compensate for the absence of real-user feedback in this study, further research should integrate patient-centered outcomes such as comprehension level, reading experience, learning efficiency, and behavioral adherence, forming a multi-dimensional evaluation system combining objective metrics and subjective patient experience. Finally, considering the decoupled relationship between text quality and readability identified in this study, optimized LLM generation strategies and adaptive linguistic polishing schemes should be developed for different medical content categories. Constructing personalized, patient-tailored AI health education frameworks will help promote the standardized, safe, and intelligent application of large language models in clinical popular science education.

## Conclusion

This study compared five LLMs for generating herpes zoster patient education texts and identified significant inter-model differences in content quality, educational suitability, and readability. Model type predominantly determined overall text quality. Readability was independently influenced by both model type and content category, with content category mainly regulating readability while exerting only limited effects on quality. Quality and readability were weakly correlated, confirming their relative independence and correcting the misconception that higher readability indicates superior educational quality.

However, these findings are constrained by several inherent limitations, including limited question sampling, undocumented model generation settings, formula-only readability evaluation, nonstandard C-PEMAT-P raw scoring, and absent verification of factual accuracy and clinical safety. Accordingly, the current results cannot support the standalone clinical application of any evaluated LLM.

Future studies should adopt standardized scoring, traceable generation protocols, repeated sampling, clinical validation, and patient comprehension testing to establish rigorous evaluation criteria for AI-generated health education materials.

## Data Availability

The raw data supporting the conclusions of this article will be made available by the authors, without undue reservation.

## Footnotes

1
http://readabilityformulas.com/
