# Supplementary material for: Artificial intelligence chatbots as information sources for herpes zoster: an evaluation of patient-education suitability, overall quality, and readability
Source: Front Public Health. 2026 Oct 27;14:1961492. doi: 10.3389/fpubh.2026.1961492 (PMC13748341; doi:10.3389/fpubh.2026.1961492)
Supplement: Supplementary file 1 [file Table_1.DOCX]

**Supplementary Table S1. Exploratory pooled Spearman correlations between assessment scores and readability indices**

| **Readability index** | **C-PEMAT-P raw total, ρ** | **GQS, ρ** |
| --- | --- | --- |
| ARI | −0.221 | −0.381 |
| FRES | 0.122 | 0.283 |
| GFOG | −0.153 | −0.317 |
| FKGL | −0.178 | −0.339 |
| CL | −0.078 | −0.234 |
| SMOG | −0.186 | −0.344 |
| LW | 0.142 | 0.255 |

Coefficients are descriptive pooled estimates across 100 responses. No inferential P values are reported because observations are clustered within questions and the pooled coefficients do not adjust for model differences. The correlation between C-PEMAT-P raw totals and GQS was ρ=0.853.
